# PET and SPECT Specialty Grand Challenge. When Knowledge Travels at the Speed of Light, Photons Take to the Field

**DOI:** 10.3389/fnume.2021.671914

**Published:** 2021-04-22

**Authors:** Alberto Cuocolo, Mario Petretta

**Affiliations:** ^1^Department of Advanced Biomedical Sciences, University Federico II, Naples, Italy; ^2^Department of Nuclear Medicine, Scientific Institute for Research and Health Care IRCCS SDN, Naples, Italy

**Keywords:** PET, SPECT–single photon emission computed tomography, diagnosis, risk stratification, machine learning

The field of nuclear medicine has been involved in a significant technological advancement and in a tremendous progress in radiochemistry that opened up new and fascinating diagnostic and therapeutic opportunities. The leap forward of stand-alone systems beside hybrid imaging designs improvement and novel theragnostic methods development made possible a wider use of nuclear medicine techniques on several diagnostic scenarios. Positron emission tomography (PET) and single-photon emission computed tomography (SPECT) are the leading nuclear medicine modalities.

## Historical Notes

These techniques have their roots not too far into the past. Among the pioneers of nuclear medicine, Dr. David E. Kuhl (born October 27, 1929 St. Louis, Missouri, died May 28, 2017 in Ann Arbor, Michigan) is known as the “Father of Emission Tomography.” This scientist has the huge merit to have led the research group who developed the SPECT camera (namely Mark II SPECT series). His group produced the world's first tomographic images of the human body in 1964, preceding by about a decade the Godfrey Hounsfield's development of the X-ray CT in 1972. The subsequent improvement of various diagnostic imaging devices, including PET, owes a lot to his pioneering research. In agreement with the original vocation of nuclear medicine, SPECT has emerged as a highly effective technology for tracing numerous functional aspects of the body such as vascular flow, metabolism, and neural transmission. PET technology has undergone a more rapid and intense development since the 1980s. This acceleration is due to two main aspects, by means the even more advantageous features of device in terms of temporal and spatial resolution and the high effectiveness of ^18^F-fluorodeoxyglucose (FDG) as marker of metabolic activity of cancerous tissue in the era of cancer research. One would in fact wonder if it is PET that decreed the success of the ^18^F-FDG or the opposite.

## Relative Merits of Pet and SPECT

It has long been discussed, and still is debated, which technique between PET and SPECT is superior in research and in the clinical arena. In 1986 Strauss and Elmaleh commented that “the future preponderance of each technique will be determined by its ability to provide timely information required for important clinical decisions” (1). Indeed, both methods will be necessary also in the future due to different strengths and limitations. One strength of SPECT is the availability and the lower costs. PET is superior due to better resolution, more sophisticated tracers, not only FDG and because it is quantitative, and absolute quantification with SPECT is a limitation as compared to PET. However, it is very likely that both techniques will still be used for different indications.

The strong interplay between research on disease development mechanisms and on organ damage identification is the key element of the recipe for success of these nuclear medicine tools. Several applications in oncology, cardiology and neurology are expressed by the use of PET and SPECT imaging. In addition, different physio-pathological patterns of systemic diseases can be investigated by both methods. In particular, the theragnostic approach to oncological patients (2, 3) finds in the use of novel tracers and advanced scanners its natural aptitude and its reason to be. Instead, cardiology has the great merit to have driven and directed the development of solid-state gamma cameras (4) that are now the big asset of all nuclear medicine field. With regard to neurology, a key role for the use of PET and SPECT scanners derives from the huge effort in radiopharmaceutical research with introduction of several imaging probes tracing specific receptor activities (5) at the service of the more fascinating and unexplored medicine field.

## Technologies that Matter

The success of these instruments is amply demonstrated by the literature and by the significant diagnostic improvement that the use of these devices has made possible both in terms of accuracy and time to reach diagnosis. This potential is further highlighted by the continuous push of research on industry for technological implementation. In particular, we have seen the realization of two strands. On the one hand, it has been introduced the conception of dedicated gamma cameras that would allow faster acquisition times with a lower dose of administered radiation and greater comfort for the patient (6, 7). These advances have materialized in hardware lighter in design and in size, accommodating large amount of data acquired in list-mode. On the other hand, we assisted to the integration of stand-alone nuclear medicine methods with morphological techniques in hybrid devices. The resourcefulness of nuclear medicine instruments is documented not only from a more strictly technical point of view with reference to the devices used but also with respect to the radiopharmaceuticals used.

In fact, the improvement of the acquisition capacity related to different photon emission spectra has made possible the simultaneous acquisition of data concerning the decay of different radionuclides at the same time, paving the way for the development of acquisition techniques with dual tracer administration to investigate different pathophysiological patterns in a single acquisition. This opportunity together with the continuous introduction of new molecules with specific diagnostic targets for the development of molecular probes that are increasingly personalized on the patient, provided several applications in oncology, neurology and cardiology as well as in the study of systemic disease with multi-organ involvement at different levels. On the other hand, in addition to the growth of the nuclear medicine tools potential, interdisciplinary knowledge at the service of imaging have further amplified the diagnostic and prognostic rule of acquired data.

The artificial intelligence approach is one of the big challenges of future imaging (8, 9). Next generation experts will have to deal with this powerful application. In other words, we are approaching an era where every single acquired pixel can be the key to read the past, the present and the future of each disease, and even when we still do not have instruments to understand data at our disposal, the very near future may offer novel opportunities to give meaning to information obtained in the past. In this context, PET, SPECT and hybrid modalities may greatly benefit of interdisciplinary knowledge to improve their diagnostic, prognostic and theragnostic potential but, at the same time, need to be ready to the new era challenges providing faster, more accurate and non-invasive methods to get as many row data as possible in a very short time at the lowest possible radiation exposure.

## Specialty Pet and SPECT Section: the Importance of Being Photon

Therefore, with the above considerations in mind, we strongly hope that the PET and SPECT section of Frontiers in the Nuclear Medicine Journal will play a central role in promoting the timely exchange of scientific information between nuclear medicine specialists and also among physicians who are called to deal with the various pathologies in which these techniques are of fundamental help. The reasons to choose this Journal are many-fold. Strengths and limitations of these two modalities are continuously changing in the context of ongoing hardware and software development and they need to be constantly understood and updated. It becomes clear that the potential overcoming of the historical limitations of each technique need to be highlighted with appropriate resonance. At the same time any improvement in terms of image reconstruction algorithms has to be made available for the scientific community so that all researchers may apply innovative tools to state-of-the art scanners. Finally, given that these technologies are the means to advance molecular imaging and have a direct impact on clinical and research practice to influence the future of molecular medicine, the main goal of this section will be promotion of knowledge exchange for prevention, diagnostics, and therapeutic strategies. Moreover, in the special issues we propose to address, with the help of authoritative experts, the possibilities of PET and SPECT in characterizing the molecular and cellular pathophysiology of various pathologies, presenting the diagnostic and prognostic value of nuclear imaging as well as verifying how PET and SPECT modify the management of patients, improving their quality of life and prognosis.

## Conclusions

The great challenge of publications in the field of nuclear medicine will be to provide an open source of robust knowledge regarding the new challenges of imaging with regard to novel acquisition systems (even smaller and more manageable in appearance and even larger with regard to the amount of data they can contain), novel radiopharmaceutical probes concerning different diagnostic and therapeutic targets, and the use of artificial intelligence tools to look further with new eyes but strong fundamentals. The contribution of scientific community will be the most valuable ally in this grand challenge.

## Author Contributions

All authors listed have made a substantial, direct and intellectual contribution to the work, and approved it for publication.

## Conflict of Interest

The authors declare that the research was conducted in the absence of any commercial or financial relationships that could be construed as a potential conflict of interest.
